# Pooled PCR testing of dried blood spots for infant HIV diagnosis is cost efficient and accurate

**DOI:** 10.1186/s12879-019-3767-z

**Published:** 2019-02-11

**Authors:** Cari van Schalkwyk, Jean Maritz, Gert U. van Zyl, Wolfgang Preiser, Alex Welte

**Affiliations:** 1 The DST/NRF Centre of Excellence in Epidemiological Modelling and Analysis (SACEMA), Stellenbosch University, Stellenbosch, South Africa; 2 Division of Medical Virology, Department of Pathology, Stellenbosch University, Cape Town, South Africa; 3 PathCare Reference Laboratory, Cape Town, South Africa; 4 National Health Laboratory Service, Cape Town, South Africa

**Keywords:** Infant, Diagnosis, Human immunodeficiency virus (HIV), Polymerase chain reaction (PCR), Cost analysis

## Abstract

**Background:**

Access to qualitative HIV PCRs for early infant diagnosis (EID) is restricted in resource-limited settings due to cost. We hypothesised that pooling of dried blood spots (DBS), defined as combining multiple patient samples in a single test with subsequent individual testing of positive pools, would be cost saving while retaining clinical accuracy compared to individual patient testing.

**Methods:**

Cost savings: A model was developed to simulate reagent and consumable cost saving of pooled compared to individual sample testing. Daily sample/result data of a public health laboratory in South Africa were used to illustrate outputs from the model. Samples were randomly allocated to pools and the process was repeated 1000 times to measure variation in estimates due to this stochasticity.

Clinical accuracy: 1170 patient samples were tested using the Roche CAP/CTM Qual assay in pools of five 50 μl DBS. Negative pools comprised DBS previously tested in single reactions; positive pools included 1 positive sample.

**Results:**

Pooling would have saved 64% of laboratory costs in 2015. The model is published as an R-based web tool, into which the user enters sample/positivity estimates and workflow management parameters to obtain cost saving estimates at an optimal pool size.

Sensitivity of pooled testing was 98.8% overall; 100% for strongly reactive pools. One pool tested false positive which would not impact clinical specificity as individual patient testing is performed prior to reporting.

**Conclusions:**

Pooled PCR testing for EID remains accurate and dramatically reduces costs in settings with moderate to low prevalence rates and sufficient sample numbers.

**Electronic supplementary material:**

The online version of this article (10.1186/s12879-019-3767-z) contains supplementary material, which is available to authorized users.

## Background

Early infant diagnosis (EID) of HIV infection in HIV-exposed infants currently requires polymerase chain reaction (PCR) to identify and treat HIV-infected infants promptly and thereby improve their prognosis [1]. However, PCR testing for EID can be prohibitively expensive in resource limited settings where the burden of disease is significant, limiting access to care [2]. Strategies to increase EID testing coverage are thus required.

The World Health Organization (WHO) recommends PCR testing for all HIV-exposed infants at 4–6 weeks of age using EDTA whole blood, plasma or dried blood spots (DBS) [3], while the importance of testing infants at birth has recently been emphasised [4]. Reducing the cost of EID testing would enable programmes to include PCR testing at additional timepoints, e.g. at birth or later during infancy, without increasing the total cost. Timely diagnosis and treatment initiation would in turn further curb HIV-related morbidity and mortality in this vulnerable population.

A pooled EID testing algorithm involves the PCR screening of a specimen pool comprising multiple individual patient specimens, followed by individual testing (referred to as pool deconvolution) only if a pool screens positive. As all individual samples in a negative pool are regarded as negative it results in substantial cost savings when a large proportion of pools tests negative. This approach is efficient in blood donor screening [5] and in HIV treatment failure detection by quantitative HIV-1 RNA testing of both plasma [6] and DBS specimens [6, 7].

In this study we demonstrate, through modelling, the cost efficiency of pooled EID testing at varying HIV positivity and sample throughput rates, as well as the clinical accuracy of pooled EID testing compared to individual patient whole blood EID testing in a public health laboratory in Cape Town, South Africa.

## Methods

### Study design and ethical approval

This was a retrospective, laboratory-based study performed at the Division of Medical Virology, Department of Pathology, National Health Laboratory Service (NHLS) Tygerberg and Stellenbosch University. Ethical approval was obtained from the Health Research Ethics Committee of Stellenbosch University with reference number N14/02/013.

### Contextual analysis

In order to assess specimen flow and positivity rate in the abovementioned laboratory, we queried routine PCR data from the Disa*Lab laboratory information system (Laboratory System Technologies (Pty) Ltd., Bedfordview, South Africa). Routine HIV PCR data was obtained by extracting the following parameters from the local Medical Virology NHLS database: unique laboratory reference number, patient name and surname, patient date of birth, sample processing date, sample result. Patient records were linked using LinkPlus software (CDC, Atlanta) to determine the final HIV status of presumed low positive samples, where after all records were de-identified.

### Reference samples used for pooling evaluation

Per laboratory standard operating procedure, the laboratory routinely stores residual EID samples as 50 μl DBS after routine diagnostic testing using 100 μl EDTA whole blood on the Roche Cobas AmpliPrep/Cobas Taqman HIV-1 Qual (CAP/CTM, Roche Molecular Systems, Inc., Branchburg, NJ) assay has been completed. The Roche CAP/CTM is a total nucleic acid real-time PCR assay that detects HIV-1 proviral DNA and HIV-1 RNA [8]. The assay is suited for EID testing in high burden areas as it is a high-throughput, automated system, which can test both whole EDTA blood and DBS samples.

Stored DBS are identified by unique laboratory reference numbers. Previously confirmed negative DBS, tested in single reactions with the reference PCR method, were combined to constitute negative pools. Positive pools were constituted by combining one DBS from a patient with a positive reference result with negative dried blood spots. A subset of the positive pools were based on ‘low positive’ samples, i.e. samples initially categorised as indeterminate (CAP/CTM cycle threshold values ≥32 and/or relative fluorescence values ≤5) but who were proven to be HIV infected at later time points. This was necessary as patients with such low positive results were previously shown to have a reduced probability of testing positive at a later time point [9, 10].

### Pre-analytical DBS manipulation

Due to effective prevention of HIV mother to child transmission (PMTCT) programmes, the prevalence of HIV infection in young infants in South Africa is now low [2]. With a very low prevalence the optimal theoretical pool size is large as most pools would remain negative, realising maximal cost savings for larger pools. Apart from the theoretical optimum we included other considerations: 1) the maximum number of DBS which could fit into a single reaction tube, 2) whether DBS could be added to a Roche S-tube directly or whether elution of DBS should be done as additional step without resulting in reaction inhibition (Roche specimen pre-extraction reagent [SPEX] was used as DBS eluant throughout), and 3) the sensitivity of pooled HIV PCR testing.

### Pooled testing model

A model was developed to simulate yearly cost savings of a pooled testing approach compared to individual DBS testing. Daily sample and result data of the NHLS Medical Virology laboratory for the period 1 January 2009 to 31 July 2015 were used for the simulation (Additional file 1). The model assumed a minimum batch size of 10 samples for individual testing and 20 samples when pooling (i.e. between 4 and 10 pools at different pool sizes), to maintain a good turn-around time while maintaining batches of adequate size to justify the use of the instrument and laboratory personnel time. Remaining samples after minimum batch sizes were filled were added to the following day’s runs. For the observation period, the model simulated the number of positive pools when randomly allocating samples tested on a particular day to varying pool sizes from 2 to 5 with deconvolution of positive pools by individual testing, done the following day. This random allocation was repeated 1000 times, for each pool size, to estimate the mean values and variability in model output. Bootstrap confidence intervals around mean estimates are given by the 2.5th and 97.5th percentiles of the 1000 estimates.

Only the cost of reagents and consumables is considered. Labour costs and laboratory overheads, such as electricity and equipment costs, are not considered.

The model to estimate cost-efficiency of the pooling approach was implemented in the statistical software R [11] and a web-based tool was created using the Shiny package, accessible at: https://carivs.shinyapps.io/Calculator/. The user can enter average daily sample throughput, workflow management parameters, expected infant HIV positivity rate and reagent costs in US dollar. The model produces estimates of costs and batched runs saved at the optimal pool size, and a plot of cost savings as a function of pool size and positivity.

## Results

### Practical evaluation of different DBS pooling approaches

Adding more than two whole DBS directly into an S-tube is not practical as DBS would not adhere to the side of the tube, thereby mechanically obstructing the CAP/CTM aspiration probe. After experimenting with DBS cut into quarters, halves or kept whole, it was found that a maximum of 5 dB, each cut into halves, could fit into a 2 ml Eppendorf tube together with 1450 μl SPEX before continuously mixing the tube at 56 °C for 30 min (the current standard practice for individual DBS testing). The resulting eluate of approximately 1100 μl was then transferred to an S-tube by manual pipetting.

The time taken to process DBS specimens for individual testing was measured and compared to time taken to prepare a similar number of DBS specimens for pooled testing, and was found to be similar.

### Clinical accuracy of pooling

A total of 1170 unique patient specimens were used to construct 149 positive, 18 low positive and 67 negative pools (Table 1). All positive pools tested positive and all but one of the negative pools tested negative. This resulted in a pool level specificity of 98.5%; the expected specificity at patient level would be higher as each sample contributing to a positive pool must be re-tested individually prior to reporting.

**Table 1:** Clinical performance of pooled testing compared to Roche CAP/CTM reference results

Pooled samples result, n (%), 5 samples per pool				
		Positive	Negative	Total
Whole blood reference result	Positive pools	149 (100)	0	**149**
	Low positive pools	16 (89)	2 (11)	**18**
	Negative pools	1 (1.5)	66 (98.5)	**67**
	Total	**166**	**68**	**234**

Two of the low-positive pools did not test positive, resulting in an overall sensitivity of 98.8% in the test population. However, for one of the two patients a second DBS of the indeterminate sample was available to be re-tested individually, with a negative result. This suggests a very low HIV viral load, which was only detected when using the larger (100 μl) whole blood input volume, so in this case pooling would not have had any clinical impact compared to individual DBS testing.

### Application of the cost-efficiency model simulation to laboratory data

From January 2009 to July 2015, the Medical Virology laboratory received an average of 45 samples per day with an overall positivity rate of 5.7% (Table 2). The observed increase in overall sample positivity observed from 2013 to 2014 coincides with a change in infant testing policy rather than a failure of PMTCT strategies. Since 2014, all positive infant PCRs are recommended to be confirmed by a second qualititative HIV-1 PCR rather than the previously recommended HIV RNA load test.

**Table 2:** Daily average specimen numbers for EID testing as well as cost savings at varying pool sizes, per annum, Division of Medical Virology, National Health Laboratory Service Tygerberg and Stellenbosch University

Year	Mean (SD) daily number of samples	Positivity rate (%)	% Cost saved (95% CI)		% Batched runs saved (95% CI)	
			Pool size = 2	Pool size = 5	Pool size = 2	Pool size = 5
2009	39 (26)	10.0	32 (31.7–32.3)	41 (40.1–42)	32 (31.2–32.8)	29.3 (27.7–31)
2010	35 (17)	9.0	34 (33.7–34.3)	44.3 (43.3–45.2)	31.1 (30.3–31.9)	28.8 (27–30.6)
2011	36 (18)	6.8	39.6 (39.4–39.8)	52.8 (52–53.5)	31.1 (30.4–32)	32.2 (30.7–33.8)
2012	40 (17)	5.1	43.1 (43–43.3)	59.7 (59.2–60.3)	37.2 (36.6–37.9)	40.4 (39.2–41.5)
2013	48 (21)	3.6	44.2 (44.1–44.4)	64.3 (63.9–64.7)	40 (39.5–40.4)	51.2 (50.4–52.1)
2014	55 (19)	4.3	42.6 (42.5–42.7)	61 (60.6–61.5)	39.8 (39.3–40.3)	50.9 (49.8–51.9)
2015	67 (26)	3.6	43.3 (43.3–43.5)	63.5 (63.1–64)	40.8 (40.4–41.3)	54.5 (53.3–55.7)
Overall	45 (23)	5.7				

*SD* Standard deviation, *CI* Confidence interval

**Fig. 1:**
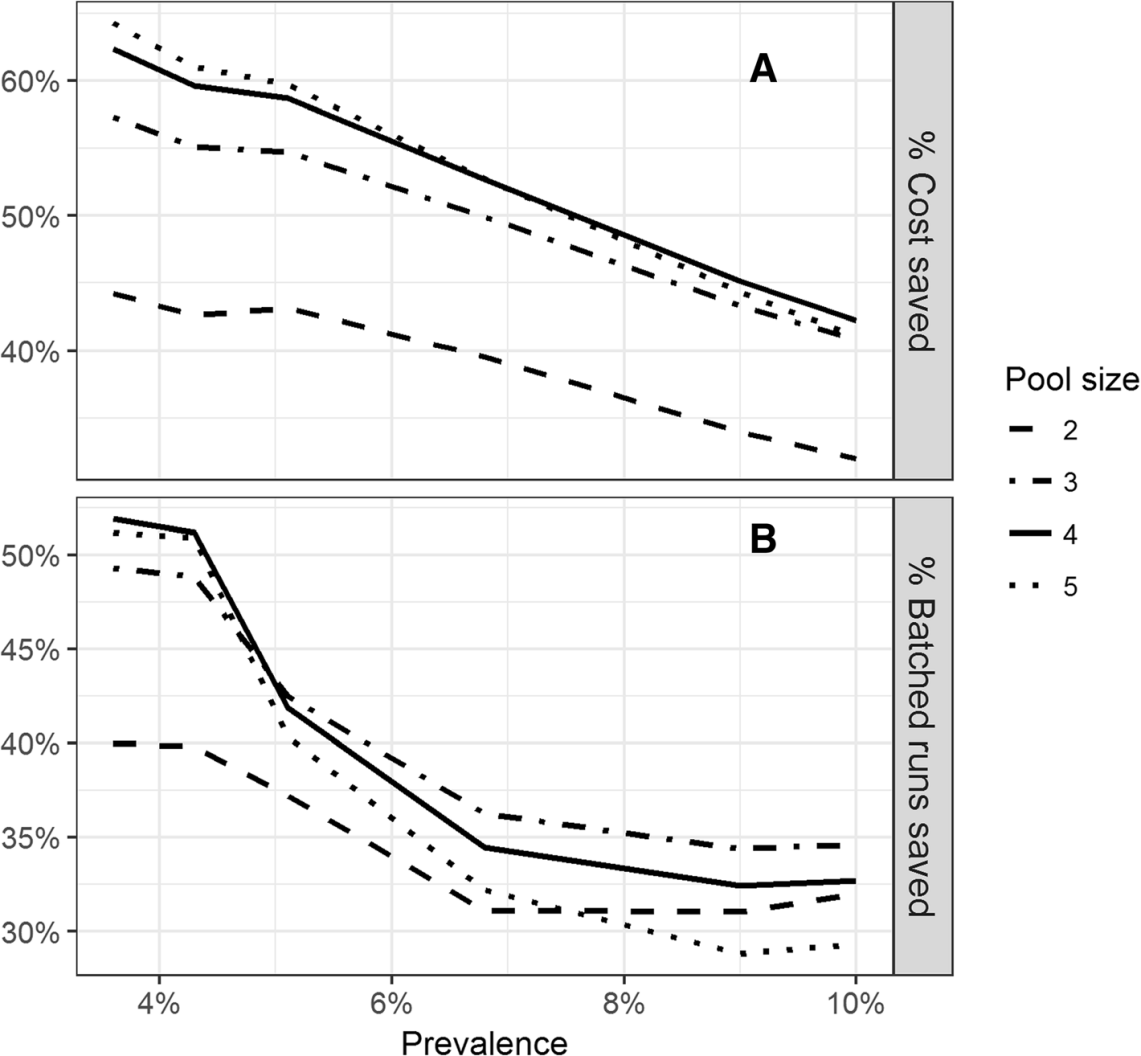
**a** Estimated % reagent and consumable cost saving at varying pool sizes and positivity rates, **b** Estimated % of batched testing runs saved at varying pool sizes and positivity rates

Figure 1a shows the percentage cost that would have been saved each year if this lab used pooling instead of individual testing. In 2015, the expected savings would have been 43.3% (95% CI, 43.3–43.5%) of reagent and consumable costs if two samples were pooled and up to 63.5% (95% CI, 63.1–64%) if five samples were pooled. Figure 1b shows the percentage of batched runs saved when using a pooling approach compared to individual testing. From 2013 to 2015 this laboratory could have saved half of the batched runs had it used pool sizes of three to five instead of individual testing.

## Discussion

With this study, we modelled the cost efficiency of pooled EID testing at varying HIV PCR positivity rates and estimated the real-life clinical accuracy of pooled EID testing compared to individual patient whole blood EID testing in a public health laboratory in Cape Town, South Africa.

In order to assess the impact of pooling over a longer period we performed a simulation using real daily individual test results. We established that pooling could reduce reagent and consumable related costs by 63.5% in our setting, which has a low expected rate of positive laboratory results. In addition we created a useful tool for laboratory managers to estimate savings and predict the optimal pool size for EID testing based on the user’s local HIV PCR result data. It is important to note that expected laboratory PCR positivity rates should be used in the estimation and *not* the expected population prevalence of infant HIV infection, as an individual patient may have two consecutive positive PCR tests as a confirmation of HIV status which will influence the efficiency of pooling. While personnel time spent to prepare pooled runs compared to individual DBS runs was similar, the median number of runs per day can be reduced through pooling. In settings where diagnostic service bottlenecks result from limited instrument availability, pooling may therefore relieve such bottlenecks and result in an improved turnaround time from sample acquisition to result reporting. A reduction in the number of runs required may in addition allow instruments to be used for other critical tests, such as HIV viral load testing. With the online tool, users further have the ability to set the minimum number of samples that will be required to perform pooled testing to individualise this process for their setting. Using the most recent UNAIDS estimates of the final mother-to-child transmission rates in 21 sub-Saharan African countries [2], a laboratory in a setting like South Africa with an estimated transmission rate of 4% and a moderate throughput of 70 specimens per day would save 62% of reagent and consumable costs using pools of 5 specimens while reducing the number of instrument batched runs from 3 to 2. Similarly, in a laboratory in a high volume, high burden setting that performs 130 tests per day at a MTCT rate of 9%, testing pools of 4 specimens would save 44% of costs and reduce the number of batched runs from 6 to 4. This may have a significant impact on the turnaround time of patient result reporting as it would allow all specimens to be tested in an 8 h work day if the Roche CAP/CTM platform is used without the need for additional instrumentation or extended working hours.

EID pooling using 50 μl DBS was importantly found to have an acceptable clinical sensitivity and specificity compared to testing 100 μl whole EDTA blood. Of the 18 pools consisting of one low positive DBS and four negative DBS, pooling failed to pick up two. Although it is important to detect samples with low HIV RNA levels, we could not include more such samples due to the relative shortage thereof. These samples comprised approximately 11% of the positive DBS pools in the study population, whereas only approximately 0.5% of all infant PCRs conducted in the investigating laboratory were reported in this category. The impact of a reduced sensitivity for this result category when using pooled testing is therefore limited, and the actual sensitivity when pooling all routine PCR samples is expected to be greater than the observed 98.8% sensitivity. It is further important to note that pooled DBS testing was compared to higher volume individual whole EDTA blood reference results, and a direct comparison between pooled DBS testing and individual DBS testing may have had even more favourable results. In the single case where a second stored DBS was available, individual DBS testing also reported a negative result.

Regarding the specificity of pooled testing, one of 68 expected negative pools were reported as positive. We previously reported a reduced specificity of the CAP/CTM assay [9], and the required deconvolution of all positive pools implies that all patients from a positive pool would receive individual DBS testing. Pooling therefore does not compromise the test’s specificity compared to individual DBS testing.

The main limitation of pooled EID testing is an increased turnaround time for positive samples (as these would require deconvolution and subsequent individual testing) as well as samples that are too few to complete a pool on the day of receipt which will be included in the next day’s testing. We therefore advise that the online tool be used to determine whether a site’s anticipated sample numbers and positivity rate would realise significant savings.

## Conclusion

EID pooling proved to be a cost-efficient alternative to individual patient testing. Costs saved by introducing pooled testing could be utilised to improve the coverage and/or frequency of EID testing, or could be used to improve other components of an HIV programme.

## Additional file

Additional file 1: NHLS Tygerberg data. This file contains the daily number of infant blood samples received at this lab and the number testing HIV positive between January 2009 and July 2015. (CSV 50 kb)

## Abbreviations

CI: Confidence interval
DBS: Dried blood spots
EID: Early infant diagnosis
HIV: Human immunodeficiency virus
NHLS: National Health Laboratory Service
PCR: Polymerase chain reaction
PMTCT: Prevention of HIV mother to child transmission
